# Diagnostic Dilemma for Low Viremia with Significant Fibrosis; is Hepatitis B Virus DNA Threshold Level a Good Indicator for Predicting Liver Damage?

**DOI:** 10.4274/balkanmedj.2017.0888

**Published:** 2018-07-24

**Authors:** Ercan Yenilmez, Rıza Aytaç Çetinkaya, Ersin Tural

**Affiliations:** 1 Department of Infectious Diseases and Clinical Microbiology, İstanbul Sultan Abdulhamid Han Training and Research Hospital, İstanbul, Turkey; 2 Department of Pediatrics, İstanbul Sultan Abdulhamid Han Training and Research Hospital, İstanbul, Turkey

**Keywords:** Chronic hepatitis B, fibrosis, HBV DNA, prediction, viremia

## Abstract

**Background:**

The most important difficulties about management of hepatitis B are still determining the liver damage and the right time to start antiviral therapy.

**Aims:**

To reveal the role of hepatitis B virus DNA threshold level for prediction of liver fibrosis and inflammation in young-aged hepatitis B e-antigen negative chronic hepatitis B patients.

**Study Design:**

Diagnostic accuracy study.

**Methods:**

A total of 273 hepatitis B e-antigen negative young chronic hepatitis B patients with any hepatitis B virus DNA levels between 2008 and 2016, who had liver biopsy after at least 6 months follow up period, enrolled in this retrospective study. We created two groups as case and control, cases with hepatitis B virus DNA levels below 2000 IU/mL and controls with hepatitis B virus DNA levels over 2000 IU/mL. Having histological activity index ≥4 or/and fibrosis scores ≥2 were defined as significant histological abnormality. Then, we analyzed the relationship between these groups.

**Results:**

We showed that significant fibrosis may occur in one third of young chronic hepatitis B patients with low viremia (30.2%, n=42/139 in cases, 55.2%, n=74/134 in controls). Among the 42 cases with low viremia and significant fibrosis, 21.4% had alanine aminotransferase level between 40-59 U/L, 42.8% had alanine aminotransferase level between 60-79 U/L, and 35.7% had alanine aminotransferase level over 80 U/L. There was weak correlation between hepatitis B virus DNA threshold level and fibrosis score (p<0.001, rho=0.253). The optimum serum hepatitis B virus DNA threshold level in our study for predicting significant fibrosis was 1293 IU/mL (p<0.001, AUC: 0.657±0.034). The optimum alanine aminotransferase threshold level for predicting significant histological activity index and fibrosis was 64.5 and 59.5 U/L, respectively. The sensitivity and the specificity of 1293 vs 2000 IU/mL hepatitis B virus DNA threshold with 60 U/L alanine aminotransferase threshold level for predicting F≥2 fibrosis score were similar (sensitivity: 0.43 and 0.38, specificity: 0.76 and 0.77, respectively).

**Conclusion:**

Significant fibrosis may occur even in young cases with low viremia. It is not possible to define a single threshold hepatitis B virus DNA level for differentiating inactive carriers from patients with hepatitis B e-antigen-negative chronic hepatitis. Diagnostic accuracy of hepatitis B virus DNA with alanine aminotransferase thresholds for the prediction of significant fibrosis is weak.

Approximately 240 million people are diagnosed with chronic hepatitis B (CHB) infection worldwide (1). CHB infection has various clinical manifestations, which can be categorized into four phases according to hepatitis B e-antigen (HBeAg) status, hepatitis B viral load, and serum alanine aminotransferase (ALT) level (2,3). However, gray zones exist, which means that HBeAg status, viral load, and transaminase level do not fall into the same phase (2). The terminology, monitoring, evaluation, and treatment criteria for CHB infection may differ slightly among the leading associations of liver diseases, such as the American Association for the Study of Liver Diseases (AASLD), the European Association for the Study of Liver (EASL) (3), the Asian Pacific Association for the Study of Liver (APASL), and the World Health Organization (WHO) (WHO, 2015 guideline for chronic hepatitis infection) (4) particularly for indeterminate cases.

Monitoring patients with CHB and predicting liver damage remains difficult. Histopathological examination is the gold standard for determining the degree of liver damage; however, clinicians hesitate to perform liver biopsy because of the high cost, invasiveness, and risk of complications of the procedure and the associated sampling error and requirement for expert histological interpretation (WHO). Thus, scholars have developed noninvasive tests based on blood or serum indices [aspartate aminotransferase (AST)-to-platelet ratio index (APRI), FIB-4, and FibroTest^©^ commercial assay] or ultrasound principles [transient elastography (e.g.), FibroScan^©^] for indirect determination of liver damage (4).

In our military hospital, we had young military personnel and candidates for military service who were diagnosed with CHB. According to the health regulations for military service in Turkey, we performed liver biopsy to those who have CHB, although they do not meet the criteria for treatment of CHB. These regulations enable us to have a large number of liver biopsy results of CHB cases with hepatitis B virus (HBV)-DNA level below 2000 IU/mL (low viremia).

This study aims to compare the histopathological findings of young patients with HBeAg-negative CHB and low viremia to high viremia and reveal if 2000 IU/mL HBV-DNA threshold level has any significance to predict liver fibrosis and inflammation.

## MATERIALS AND METHODS

This retrospective study was conducted in a tertiary training and research hospital. The study was approved by the Institutional Ethics Committee on May 22, 2017 (HNEAH-KAEK 2017/KK/71). Data were gathered by the clinicians who were in charge of the follow-up of patients during the study period.

### Population

A total of 273 young male patients with HBeAg-negative CHB patients who were followed-up between January 2008 and December 2016 were enrolled in the study. All patients were subjected to liver biopsy after they were determined to have HBsAg-positivity and ALT level over 40 U/L for at least six months, regardless of their HBV DNA levels. Patients who had history of CHB treatment with interferon or antiviral drugs and patients coinfected with other hepatitis viruses and human immunodeficiency virus were excluded in the study. Patients had no other chronic liver diseases, such as fatty liver disease, toxic hepatitis, and autoimmune hepatitis; any symptoms of liver cirrhosis; and history of other chronic diseases and chronic drug use.

### Study design

Patients in the study were HBeAg-negative, followed-up for at least 6 months, had ALT levels over 40 U/L at the beginning and end of the follow-up period. The patients were divided into two groups, namely, case (with HBV-DNA levels below 2000 IU/mL) and control (with HBV-DNA levels over 2000 IU/mL) groups. Significant histological abnormality was defined as necroinflammation grade ≥4 and/or fibrosis stage ≥2 according to literature (5,6,7,8). We analyzed the relationship between the two groups.

### Liver biopsy and histology

Patients underwent liver biopsy with 16G biopsy needles through Menghini’s aspiration method or subcostal real-time ultrasound-guided liver biopsy by Trucut-style. A qualified biopsy specimen had length of at least 1.5 cm and more than four portal tracts. Ishak’s scoring system was used to determine histologic necroinflammation and fibrosis stages. Not all pathological assessments were conducted by the same pathologist, but all pathologists involved in the study are trained in the same pathology department and are still working in the same pathology laboratory.

### Laboratory tests

Serum biochemistry tests for ALT and AST were conducted using commercial kits. The upper limit of the normal (ULN) ALT level was set at 40 U/L. HBV serological markers were detected using chemiluminescence microparticle immunoassay (Abbot, Architect System, Germany). HBV-DNA was quantitatively determined by HBV QNP 2.0 Real-time Polymerase Chain Reaction Assay (Iontek, Turkey) with the lowest detection limit of 10 IU/mL (80 copies/mL) and limit of quantification between 2×10^9^ and 2×10^1^ IU/mL.

### Statistical analysis

Statistical analyses were performed using Statistical Package for Social Sciences version 15. The baseline characteristics were presented as means and standard deviations for continuous variables and as frequencies and percentages for categorical ones. Continuous variables were compared by independent-sample Student’s t test and Mann-Whitney U test according to their distributions. Categorical variables were compared using chi-squared test and Fisher’s exact test. Spearman’s rank correlation coefficient (rho) was used to express correlations among parameters. The receiver operation characteristic (ROC) curve was analyzed to determine the diagnostic performance of the optimal threshold levels for serum ALT and HBV-DNA. Diagnostic performance was analyzed by VassarStats statistical software (online), and comparisons were conducted by McNemar test and kappa statistic among different diagnostic performance scores for actual and recommended threshold levels of parameters. G*Power 3.1.9.2 was used for post-hoc power analysis. The study has 90% power to detect a minimum of 20% difference for significant fibrosis and hepatic inflammation among patients categorized according to their serum levels of HBV-DNA and ALT.

## RESULTS

A total of 273 patients with HBeAg-negative CHB were included in the study. All the patients were male and young and had no other accompanying chronic diseases. The mean age, ALT level, and HBV-DNA levels of all patients were 23.78±4.2 years, 89.25±80.18 U/L, and 1.4×10^7^ IU/mL±9.8×10^7^ IU/mL, respectively. The mean age was not different among histological activity index (HAI) and fibrosis groups (p=0.516 and 0.905, respectively) (Table 1). In particular, 184 (67%) patients were aged between 19 and 24 years, and 89 (33%) patients were aged between 25 and 40 years (Table 2). Eight-nine (32.6%), 73 (26.7%), and 111 (40.6%) patients had ALT levels of over 80, 60–79, and 40–59 U/L, respectively. The number of patients who had HBV-DNA level below 2000 IU/mL was 139 (51%). One hundred forty-six (53%) patients had significant HAI (≥4) score, and 116 (42%) had significant fibrosis (≥2) score. The distribution of patients did not vary across age groups in relation to HAI and fibrosis thresholds (p=0.378, p=0.962). However, the distribution of patients varied across ALT groups in relation to HAI and fibrosis thresholds (p=0.039, p=0.003, p=0.022), except for ALT threshold of 80 U/L and fibrosis (p=0.176), and across HBV-DNA groups in relation to HAI and fibrosis thresholds (p=0.003, p=0.000). The fibrosis threshold exhibited poor correlation with HBV-DNA, ALT threshold level, and HAI among all the groups analyzed (rho: <0.25) (Table 2).

**Figure 1:**
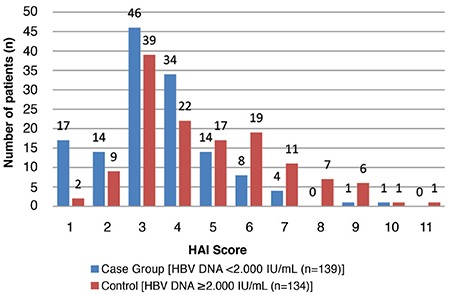
Distribution of patients in the case and control groups in relation to HBV DNA threshold level and HAI score. Numbers of patients (n) within each histology activity index score were indicated separately on the top of bars. *HAI: histology activity index; HBV: hepatitis B virus*

**Figure 2:**
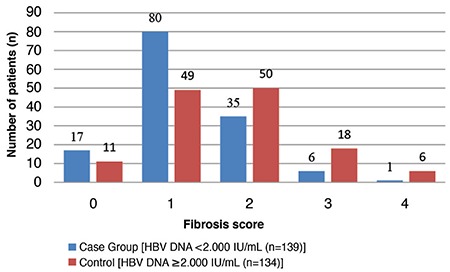
Distribution of patients in the case and control groups in relation to HBV DNA threshold level and fibrosis scores. Numbers of patients (n) within each histology activity index score were indicated separately on the top of bars. *HBV: hepatitis B virus*

The HAI scores were 1–10 for the <2000 IU/mL group and 1–11 for the ≥2000 IU/mL group; of which, most of the cases had HAI scores of 3 and 4, respectively (Figure 1). The fibrosis scores of patients in both groups varied between 0 and 4, and most of them had fibrosis scores of 1 and 2 (Figure 2). Most patients with fibrosis scores of 3 and 4 belonged to the ≥2000 IU/mL group.

In the <2000 IU/mL HBV-DNA group, 44.6% (n=62/139) of the patients had significant HAI scores, and 30.2% (42/139) had significant fibrosis scores. In the ≥2000 IU/mL group, the ratios of the cases with significant scores were 62.7% (n=84/134) for HAI and 55.2% (n=74/134) for fibrosis (Table 2).

When we searched for the ALT threshold distribution of patients particularly those with low viremia and ≥2 fibrosis, 21.4% (9/42), 42.8% (18/42), and 35.7% (15/42) patients had ALT levels of 60–79, 40–59, and over 80 U/L, respectively (Table 3).

**Figure 3:**
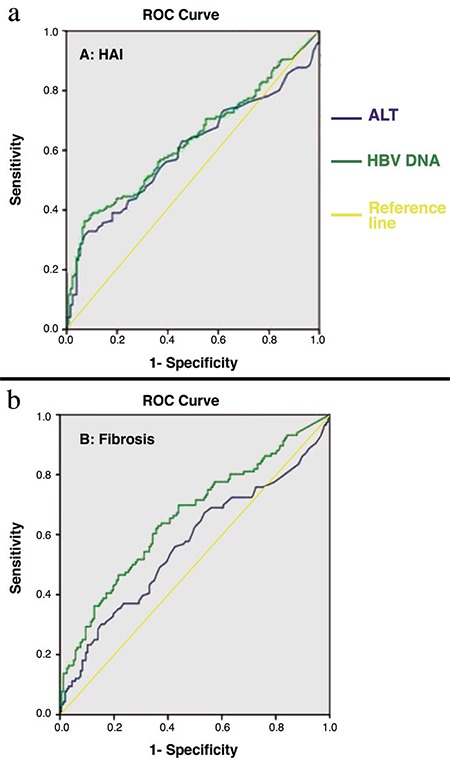
Area under the receiver operating characteristic curve of alanine aminotransferase and HBV DNA levels for predicting significant inflammation (HAI ≥4) and fibrosis (F≥2) scores. *HAI: histology activity index; HBV: hepatitis B virus*

Basing on the ROC curves of serum HBV-DNA and ALT levels, we determined the threshold levels with the optimum sensitivity and specificity for detection of HAI ≥4 and F≥2. The optimum serum HBV DNA threshold levels for our study group to predict significant HAI and fibrosis were 1102 and 1293 IU/mL, respectively. The optimum ALT threshold levels for predicting significant HAI and fibrosis were 64.5 and 59.5 U/L, respectively (Table 4 and Figure 3a, 3b).

We also analyzed the diagnostic accuracy of the HBV-DNA threshold level of 2000 IU/mL with the ALT threshold of 60 U/L and the HBV-DNA threshold level of 1293 IU/mL with the ALT threshold of 60 U/L for the prediction of significant fibrosis (F≥2). The sensitivity, specificity, positive predictive value, and negative predictive value for the HBV-DNA threshold of 2000 IU/mL were 0.38, 0.77, 0.56, and 0.63, respectively. The sensitivity, specificity, positive predictive value, and negative predictive value for the HBV DNA threshold of 1293 IU/mL were 0.43, 0.76, 0.57 and 0.64, respectively (Table 5).

## DISCUSSION

Cirrhosis and hepatocellular carcinoma (HCC) are the main complications of HBV infection; HCC with or without cirrhosis occurs primarily due to HBV in more than 50% cases in countries, such as Turkey, where HBV infection is endemic (9).

Recent guidelines for the management of HBV were published by WHO, AASLD, and APASL in 2015 and by EASL in 2017 (1,2,3,4). The terminology, monitoring, evaluation, and treatment criteria for CHB infection vary among these organization, especially with regard to indeterminate cases (gray zones). The differences among the guidelines are mostly about the HBV-DNA and ALT thresholds, biopsy indications, requirement for noninvasive methods, and treatment indications (1,2,3,4). Moreover, clear-cut definitions of histological findings of patients that need to be treated are not defined in the published guidelines and in literature. The terms “insignificant” and “significant” or “no/mild”, “moderate”, and “severe” are used to define the level of fibrosis or necroinflammation, respectively, in literature. F0 and F1 refer to no/mild fibrosis, F2 refers to moderate fibrosis, F3 or F4 refers to moderate fibrosis, and F5 or F6 refers to cirrhosis (8). In other studies, F<2 defines insignificant and ≥2 defines significant fibrosis scores (5,6,7). In the present study design, we determined F2 score as significant fibrosis. Almost all liver histology studies were based on fibrosis score, which is the most important finding and endpoint of CHB infection. Therefore, consensus for the limit of significant inflammation has not been established, and only few studies focused on HAI score in literature. Considering that most studies defined grade ≥4 as significant HAI score, we applied this criterion in the present work (5,6,7,8).

In this study, we mainly aim to reveal the histopathological findings of liver in young patients with CHB infection. Managing CHB infection is influenced by several factors, such as patient’s age, family history of cirrhosis, and HCC. As such, practical guidelines do not generally, recommended to perform liver biopsy before the age of 30 years (1,2,3,4). In the present work, given that the patient population consists of young military personnel, the mean age of the patients (23.91±0.29) who were subjected to liver biopsy was lower than the age limit in the guidelines. HBV is commonly transmitted by mother-to-child transmission in endemic countries, such as Turkey, and by sexual transmission in other countries; hence, the infection begins to take effect in very young patients in endemic countries. When patients who are infected on birth reach the age of 35-40 years, they have been already exposed to the effect of HBV infection for at least 15-20 years, which are longer than the exposure duration of patients infected with CHB by other ways. In this regard, the lifetime risk of developing end-stage liver diseases is high in endemic countries and can reach up to 40% (10). The present results showed that 90% of the patients studied had histopathological findings of liver fibrosis (F≥1), and almost all of these patients were aged between 20 and 30 years.

The WHO, AASLD, APASL, and EASL guidelines recommend the monitoring of HBeAg status, HBV DNA level, and ALT level for detection of HBV infection phase. According to these guidelines, the ALT threshold level remains controversial. The WHO and AASLD guidelines decrease the ALT threshold levels to 19 U/L for females and 30 U/L for males. By contrast, the APASL and EASL guidelines acknowledge the lack of available data to support the need for decreasing the traditional thresholds for the ULN of ALT values. At present, the traditional ALT threshold level (40 U/L) is still use in most clinical studies about CHB infection (11,12). The present work also used the traditional ULN for ALT because of the health regulations for military service in Turkey and the lack of histopathological data for patients who have persistently low ALT levels (PNALT).

The only definite indication for treatment of CHB is the cases with persistent HBV DNA levels above 2000 IU/mL and ALT levels >2 times the ULN; as such, the management of the cases must be individualized for the rest of the cases (1,2,3,4). Because of the poor correlations between ALT thresholds and HAI or fibrosis scores in the present study, we cannot rely on a particular ALT threshold for prediction of liver damage. Also, Seto et al. (13) reported that elevated ALT levels do not accurately help in predicting significant liver damage; on the other hand, according to the meta-analysis and systematic review by Nguyen et al. (14), significant fibrosis can be detected in patients with slight increases in ALT level.

AASLD and WHO recommend the use of the ALT threshold of 30 U/L and approve the criterion of two times increase in ULN (60 U/L) for detection of liver damage; meanwhile, EASL and APASL approve the ULN as 40 U/L (1,2,3,4). Our findings for ALT in ROC analylsis are more concordant with the AASLD and WHO recommendations (1,2,3,4); hence, decreasing the ALT ULN level to 30 U/L for male might be considered.

Almost all studies about histopathological findings in CHB infection recruited patients with HBV DNA over 2000 IU/mL. In Turkey, Barut et al. (15) concluded that HBV DNA ≥200000 IU/mL could predict histologically active disease with treatment indication (histological activity ≥6 and/or grade ≥2 by Ishak’s classification); however, a significant portion of the patients with HBV DNA 2000-200000 IU/mL also carried an indication for treatment. Thus, sufficient effort is suggested to find out significant liver damage. In another study on patients with HBeAg–negative CHB having HBV DNA over 2000 IU/mL, Sanai et al. (16) concluded no significant difference in using the HBV DNA threshold of 20000 versus 2000 IU/mL; moreover, low HBV DNA thresholds could be adopted with marginal gains in fibrosis detection and without loss of diagnostic accuracy. In contrast to these studies, the present work consisted of patients with HBV DNA over 2000 and below 2000 IU/mL. The number of patients with significant HAI and fibrosis scores in the high viremic group was higher than that in the low viremic group. In the low viremic group, 70% of the patients had <2 fibrosis score and they only required monitoring. When we consider this finding from another point of view, two of every three patients had significant fibrosis score in low viremic cases. When we evaluated the low viremic group in relation to ALT levels, the most important finding was that the ratio of F≥2 fibrosis increased over 60 U/L threshold level; hence, we should be more careful about these patients. Close monitoring of HBV DNA and additional noninvasive tests might be useful for management of patients with low viremia and high ALT level.

According to the systematic review of Papatheodoridis et al. (12), histologically significant liver disease is rare in patients with PNALT CHB and HBV DNA level between 2000 and 20000 IU/mL. By contrast, in another study of Papatheodoridis et al. (17) on 42 patients with mean age of 43 years and with HBV DNA level below 2000 IU/mL, 59.5% of the patients had F≥2 fibrosis score. The discrepancy in the results reported by the same author could be due to the differences in the ALT levels; hence, having normal ALT levels might be more important than having low viral loads for excluding significant fibrosis. This hypothesis was supported by our study results considering that significant fibrosis may occur in low viremic patients with high ALT. Sanai et al. (18), Kumar et al. (7) and Abdo et al. (19) reported significant fibrosis (F≥2) rates of 29.3%, 22.2%, and 19.6%, respectively, in low viremic CHB cases; these studies employed 58, 9, and 97 patients, respectively, with mean ages of 37.6, 33.6, and 38.2 years, respectively. These fibrosis rates were supported by the resuls in our study, and in contrast to these studies, the present work has the highest number of cases and the youngest patients with CHB.

Defining a single threshold of the HBV-DNA level for differentiating inactive carriers from patients with HBeAg-negative CHB is not possible due to the fluctuating course of the disease (20). Ormeci et al. (21) revealed that patients with HBV-DNA of 2000-20000 IU/mL were more likely to require treatment (F≥2 or HAI ≥6) compared with patients with viral load ≥20000 IU/mL. In their study on the relationship between HBV-DNA level and liver histological grade and stage, Shao et al. (22) concluded that serum HBV DNA level is not correlated to histological grade or stage of liver diseases. In the present study, the HBV DNA thresholds for detection of significant HAI and fibrosis were 1102 and 1293 IU/mL, respectively; but the sensitivity and specificity rates were not high.

We also discussed these thresholds from a different aspect and compared the ALT threshold of 60 U/L and the HBV-DNA threshold of 2000 IU/mL against the ALT threshold of 60 U/L and the HBV-DNA threshold of 1293 IU/mL in terms of diagnostic accuracy for prediction of significant fibrosis. We revealed that the diagnostic accuracy of these thresholds were poor, and no differences were found in specificity, positive predictive value, and negative predictive value; meanwhile, the sensitivity increased when we used the HBV DNA threshold of 1293 IU/mL instead of 2000 IU/mL.

Overall, the use of ALT and HBV-DNA thresholds alone or together may not accurately help us to predict significant liver damage. Microbiological and biochemical tests exhibit limitations for monitoring patients with CHB.

Significant fibrosis may occur even in young patients with low viremia. The duration of infection and the family history of cirrhosis or HCC must be considered for deciding treatments particularly in countries where HBV infection is endemic.

Defining a single threshold HBV-DNA level for differentiating inactive carriers from patients with HBeAg-negative CHB is impossible. Current biochemical, serological, and molecular markers are also not reliable for discriminating the real phase of HBV infection and liver fibrosis.

Liver histopathological examination should be considered for patients in early stages of life, particularly those with family history of CHB infection possibly by mother-to-child transmission, to determine the stage of disease or the need for treatment.

Thus far, few studies have focused on indeterminate cases with low viremia. In this regard, further randomized and controlled studies must be conducted for setting clear and definite recommendations to help clinicians manage these CHB cases.

## Figures and Tables

**Table 1:**
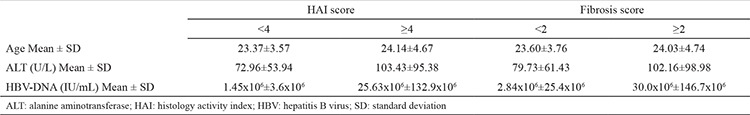
Mean age, ALT level, and HBV DNA level of patients grouped according to their HAI and fibrosis scores

**Table 2:**
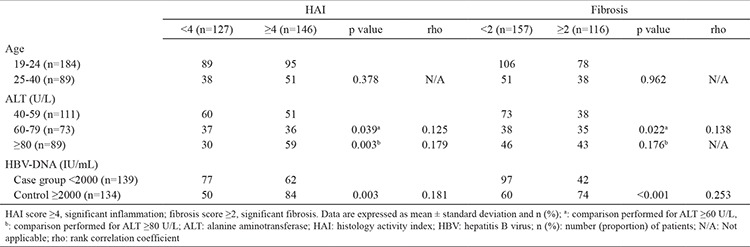
Distribution of patients according to age groups, ALT level, and HBV DNA level in relation to HAI and fibrosis scores

**Table 3:**
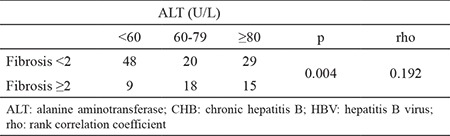
Distribution of cases according to fibrosis and ALT thresholds in low viremic (HBV-DNA <2000 IU/mL) patients with CHB

**Table 4:**
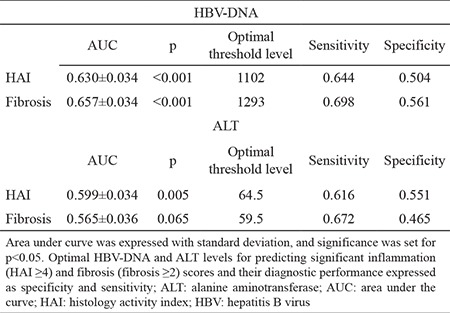
Optimal threshold levels for predicting significant HAI and fibrosis

**Table 5:**
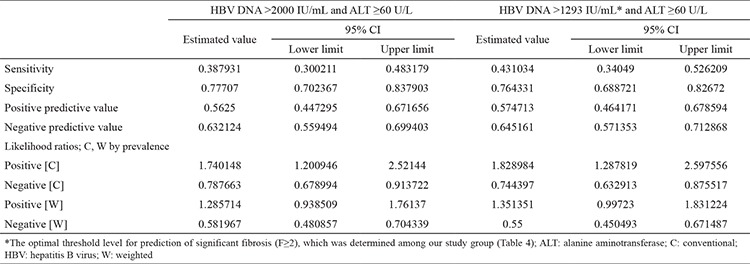
Diagnostic accuracy of the two HBV-DNA threshold levels and the ALT threshold of 60 U/L for prediction of significant fibrosis (F≥2)

## References

[1] Sarin SK, Kumar M, Lau GK, Abbas Z, Chan HL, Chen CJ, et al (2016). Asian-Pacific clinical practice guidelines on the management of hepatitis B: a 2015 update. Hepatol Int.

[2] Terrault NA, Bzowej NH, Chang KM, Hwang JP, Jonas MM, Murad MH, et al (2016). AASLD guidelines for treatment of chronic hepatitis B. Hepatology.

[3] No authors listed (2017). European Association for the Study of the Liver. Electronic address: easloffice@easloffice. eu; European Association for the Study of the Liver. EASL 2017 Clinical Practice Guidelines on the management of hepatitis B virus infection. J Hepatol.

[4] No authors listed (2015). Guidelines for the Prevention, Care and Treatment of Persons with Chronic Hepatitis B Infection. Geneva: World Health Organization; 2015 Mar. WHO Guidelines Approved by the Guidelines Review Committee.

[5] Eren M, Reyhanioglu S, Ciftci E, Dundar E, Us T, Dinleyici CE, et al (2016). Correlation of Liver Enzymes and Liver Histology in Chronic Hepatitis B Virus Infection. J Clin Anal Med.

[6] Shafaei S, Soleimani Amiri S, Hajiahmadi M, Sadeghi-Haddad-Zavareh M, Bayani M (2013). Histological grading and staging of liver and its relation to viral loads in chronic anti-HBe positive hepatitis. Caspian J Intern Med.

[7] Kumar M, Sarin SK, Hissar S, Pande C, Sakhuja P, Sharma BC, et al (2008). Virologic and histologic features of chronic hepatitis B virus-infected asymptomatic patients with persistently normal ALT. Gastroenterology.

[8] Bruden DJT, McMahon BJ, Townshend-Bulson L, Gounder P, Gove J, Plotnik J, et al (2017). Risk of end-stage liver disease, hepatocellular carcinoma, and liver-related death by fibrosis stage in the hepatitis C Alaska Cohort. Hepatology.

[9] Can A, Dogan E, Bayoglu IV, Tatli AM, Besiroglu M, Kocer M, et al (2014). Multicenter epidemiologic study on hepatocellular carcinoma in Turkey. Asian Pac J Cancer Prev.

[10] Kao JH (2014). Risk stratification of HBV infection in Asia-Pacific region. Clin Mol Hepatol.

[11] Charatcharoenwitthaya P, Phisalprapa P, Pausawasdi N, Rungkaew P, Kajornvuthidej S, Bandidniyamanon W, et al (2016). Alanine aminotransferase course, serum hepatitis B virus DNA, and liver stiffness measurement for therapeutic decisions in hepatitis B e antigen-negative chronic hepatitis B. Hepatol Res.

[12] Papatheodoridis GV, Manolakopoulos S, Liaw YF, Lok A (2012). Follow-up and indications for liver biopsy in HBeAg-negative chronic hepatitis B virus infection with persistently normal ALT: a systematic review. J Hepatol.

[13] Seto WK, Lai CL, Ip PP, Fung J, Wong DK, Yuen JC, et al (2012). A large population histology study showing the lack of association between ALT elevation and significant fibrosis in chronic hepatitis B. PLoS One.

[14] Nguyen LH, Chao D, Lim JK, Ayoub W, Nguyen MH (2014). Histologic changes in liver tissue from patients with chronic hepatitis B and minimal increases in levels of alanine aminotransferase: a meta-analysis and systematic review. Clin Gastroenterol Hepatol.

[15] Barut S, Gemici Ü, Güneş F, Demir O, Duygu F (2017). Predictors of histological indication for treatment in HBeAg negative chronic HBV infection. J Med Virol.

[16] Sanai FM, Babatin MA, Bzeizi KI, Alsohaibani F, Al-Hamoudi W, Alsaad KO, et al (2013). Accuracy of international guidelines for identifying significant fibrosis in hepatitis B e antigen--negative patients with chronic hepatitis. Clin Gastroenterol Hepatol.

[17] Papatheodoridis GV, Manesis EK, Manolakopoulos S, Elefsiniotis IS, Goulis J, Giannousis J, et al (2008). Is there a meaningful serum hepatitis B virus DNA cutoff level for therapeutic decisions in hepatitis B e antigen-negative chronic hepatitis B virus infection? Hepatology 2008;48:1451-9. 2008;48:1451-9..

[18] Sanai FM, Helmy A, Bzeizi KI, Babatin MA, Al-Qahtani A, Al-Ashgar HA, et al (2011). Discriminant value of serum HBV DNA levels as predictors of liver fibrosis in chronic hepatitis B. J Viral Hepa.

[19] Abdo AA, Bzeizi KI, Babatin MA, AlSohaibani F, AlMana H, Alsaad KO, et al (2014). Predictors of significant fibrosis in chronic hepatitis B patients with low viremia. J Clin Gastroenterol.

[20] Chu CJ, Hussain M, Lok AS (2002). Quantitative serum HBV DNA levels during different stages of chronic hepatitis B infection. Hepatology.

[21] Ormeci A, Aydin Y, Sumnu A, Baran B, Soyer OM, Pinarbasi B, et al (2016). Predictors of treatment requirement in HBeAg-negative chronic hepatitis B patients with persistently normal alanine aminotransferase and high serum HBV DNA levels. Int J Infect Dis.

[22] Shao J, Wei L, Wang H, Sun Y, Zhang LF, Li J, et al (2007). Relationship between hepatitis B virus DNA levels and liver histology in patients with chronic hepatitis B. World J Gastroenterol.

